# Parameters Influencing the Outcome of Additive Manufacturing of Tiny Medical Devices Based on PEEK

**DOI:** 10.3390/ma13020466

**Published:** 2020-01-18

**Authors:** Yiqiao Wang, Wolf-Dieter Müller, Adam Rumjahn, Andreas Schwitalla

**Affiliations:** 1 Dental Materials and Biomaterial Research, Department of Prosthodontics, School of Dentistry, Charité-University Medicine Berlin, Aßmannshauser Str. 4–6, 14197 Berlin, Germany; 2 Orion Additive Manufacturing GmbH, Gustav-Meyer-Allee 25, 13355 Berlin, Germany

**Keywords:** PEEK, fused deposition modeling (FDM), printing parameters, mechanical characteristics, medical devices

## Abstract

In this review, we discuss the parameters of fused deposition modeling (FDM) technology used in finished parts made from polyether ether ketone (PEEK) and also the possibility of printing small PEEK parts. The published articles reporting on 3D printed PEEK implants were obtained using PubMed and search engines such as Google Scholar including references cited therein. The results indicate that although many have been experiments conducted on PEEK 3D printing, the consensus on a suitable printing parameter combination has not been reached and optimized parameters for printing worth pursuing. The printing of reproducible tiny-sized PEEK parts with high accuracy has proved to be possible in our experiments. Understanding the relationships among material properties, design parameters, and the ultimate performance of finished objects will be the basis for further improvement of the quality of 3D printed medical devices based on PEEK and to expand the polymers applications.

## 1. Introduction

Polyether ether ketone (PEEK) is one of the most important members of the polyaromatic ether ketone (PAEKs), which is a family of high-performance thermoplastic polymers, consisting of an aromatic backbone molecular chain, interconnected by ketone and ether functional groups [1,2]. The molecular structures of PAEKs contain rigid benzene rings, which makes them resistant to high temperature and chemical attacks. Meanwhile, the ether bonds in the molecular backbone of polyether ketones are responsible for the flexibility of the polymers, giving them the possibility to be processed [2,3]. PEEK owns a melting point (T_m_) of 343 °C and the glass transition temperature (T_g_) of 143 °C and shows excellent mechanical properties including high strength, elastic modulus, and fracture toughness. PEEK is generally considered a high-performance thermoplastic polymer [4], and is getting increasing attention in the medical field. PEEK was commercialized by the UK company Invibio as a biomaterial for implants in 1998 because it can resist degradation in vivo [5]. Being considered as the leading thermoplastic candidate for replacing metal implant components, PEEK parts such as spine cages and retaining rings for acetabular cup assembly especially have potential in orthopedics [3,6,7] and trauma [8] due to its cortical bone-like elastic modulus.

**Figure 1:**
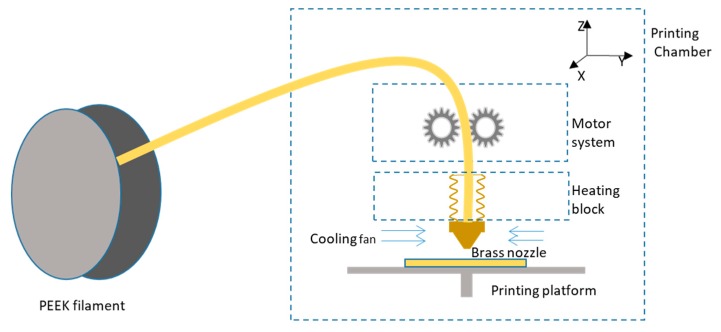
Schematic diagram of fused deposition modeling (FDM) 3D polyether ether ketone (PEEK) printer.

Many techniques are used for the production of porous PEEK for medical applications. Traditional manufacturing process methods include injection molding, extrusion, compression molding, machining, and so on [9]. Additionally, advancements in additive manufacturing (AM) continue to provide new opportunities for biomedical applications by enabling the creation of more complex architectures, e.g., for tissue engineering scaffolding and patient-personalized implants. The selective laser sintering (SLS) technique was used a decade ago in PEEK 3D printing, which is a type of powder-based AM technology. It is capable of fabricating porous PEEK-based composites with very complex architectures, permitting greater freedom of design [10]. Both 3D systems and EOS have commercialized PEEK in their SLS machines. However, the high cost and concentrated laser beam restrict it from sintering large areas or laminates. Another power-based technique is also applied in PEEK printing. In 2019, Lee et al. [11] reported their efforts toward 3D printing of PEEK by direct-ink writing technology at room temperature, which was enabled by a unique formulation comprised of commercial PEEK powder, soluble epoxy-functionalized PEEK (ePEEK), and fenchone. This combination formed a Bingham plastic that could be extruded using a readily available direct-ink write printer. Besides the techniques mentioned above, fused deposition modeling (FDM) is currently the most widely used 3D printing strategy and low-cost technology for thermoplastic materials [12]. In the FDM process, a filament is extruded from a nozzle continuously while heated to a semiliquid state, then the filament rapidly adheres with the surrounding material and solidifies, and the deposits follow a certain routine to form the desired shape [13]. The schematic of an FDM PEEK printer is showed in Figure 1. Despite a large number of publications on extrusion-based AM of porous structures using other materials than PEEK [14], there are few reports dealing with the AM of small PEEK parts, especially samples without defects such as warpage or delamination.

In this paper, we will discuss the parameters important for the AM applied to PEEK implants, based on current publications as well as our own research. We highlight the influence of process and design parameters on the performance and mechanical properties of small printed PEEK objects.

## 2. Literature Survey

Literature was obtained using PubMed and search engines such as Google Scholar including references cited therein. All articles included in this review were published within five years before October 2019. We searched for the following terms: “PEEK or Polyether ether ketone” and “3D printing or additive manufacturing” and “parameter”. We excluded review articles, case reports, and articles without detailed information on printing parameters. Only articles in English and that were highly related to PEEK processing parameters using extruded strategy were included.

## 3. Results

Thirteen articles from twelve different authors are included in our review. The detailed parameters applied in the experiments and mechanical properties of printed samples are summarized in Table 1 and Table 2.

Methods for the characterization of printed PEEK samples are mainly scanning electron microscope (SEM) [15,22,23], 3D scanner [15], differential scanning calorimetry (DSC) [13], and water contact angle measurements [23]. These methods mainly assess the dimensional accuracy, surface roughness, microstructure of interface, and crystalline ratio of printed PEEK objects.

The reviewed articles conducting mechanical tests of 3D printed PEEK show that temperature, raster angle, layer thickness, filling ratio, and printing speed are the main factors influencing the mechanical properties of printed PEEK. The maximal tensile, bending, and compression strength of 3D printed pure PEEK specimens are similar or even better than the reference as seen in Table 2. Carbon-fiber-reinforced PEEK performs as a substitute for traditionally processed PEEK [19,23,26].

**Figure 2:**
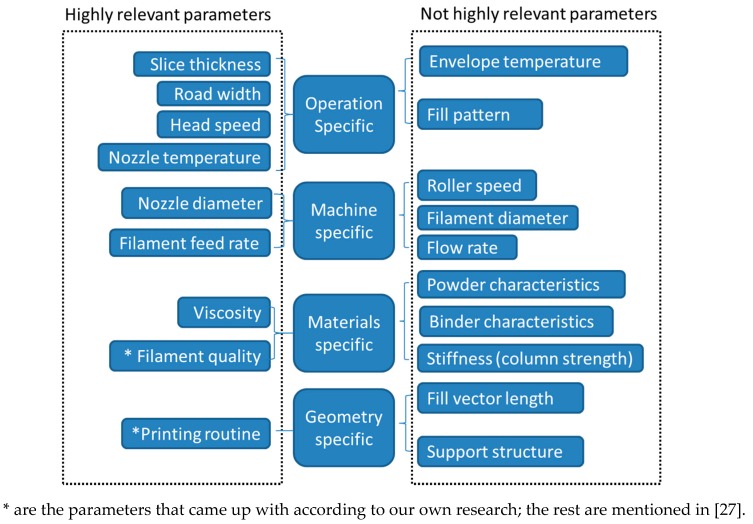
Schematic diagram of parameters that have effects on small PEEK parts made from FDM [27].

According to the literature, understanding the properties of PEEK and the settings of the printer are most important to achieve satisfying results. To expand PEEK application in dentistry, more efforts in exploring parameters need to be made. Based on the theory of Agarwala et al. [27], as well as our own researches, the most concerned parameters for printing small PEEK parts are listed in Figure 2. In the special case of PEEK printing with FDM, the not highly related parameters are either not involved, such as power characteristics and binder characteristics, or of secondary priority for small parts, such as fill pattern and support structure.

## 4. Discussion

In this part, we talk about the parameter matters for small printed PEEK objects as summarized in the schematic diagram.

### 4.1. Review of Literature and Printing Parameter Settings

#### 4.1.1. Viscosity and Specific Thermal Properties of PEEK

Some physical characteristics of PEEK related to 3D printing are shown in Table 3. PEEK, as a semi-crystalline polymer, has exceptional properties than other thermoplastic materials regarding its high T_m_ and T_g_. PEEK has crystalline and amorphous domains: the macromolecular chains in the crystalline region align in better order and own stronger intermolecular forces, causing greater strength and rigidity. The macromolecular chains in the amorphous region prefer to intertwine loosely and are easy to be scattered and stretched, showing good extensibility [28]. Generally, thermoplastics need to be heated at least beyond the T_g_ to be processed. Since reaching glass transition temperature is not enough to break the crystal lattice, the FDM processing temperature used for PEEK printing is far above T_g_, when both crystalline and non-crystalline phase are flexible. The detailed temperature for printing is explained in Section 4.1.2.

The coefficient of thermal expansion (CTE) is another factor that has an influence on PEEK printing quality. The CTE of PEEK is 5.5 × 10^−5^ K^−1^ below the temperature of 143 °C, and 14.0 × 10^−5^ K^−1^ above T_g_. Assuming printing a PEEK part with a nozzle temperature of 380 °C, it is divided into two phases, above and below T_g_. In the first phase when the material is extruded, it cools down rapidly from 380 °C to ambient temperature. During the first phase, the ΔL/L is 3.3%, which is the linear shrinkage deformation rate of the sample. During the second phase from T_g_ to room temperature (25 °C), the length deformation variance is 0.6%. Wu et al. [15] reported that the warping deformation using FDM is minimal with a chamber temperature of 130 °C and a nozzle temperature of 350 °C. In their single factor experiment, as the chamber temperature increases from 90 °C to 130 °C, the sample deformation is reduced significantly. Hu et al. [24] reported that they decreased the warpage of PEEK parts’ edge from 20.4% to 5.0% by applying higher chamber temperature, adding a heat collector module, a new heater to the nozzle, and using a PEEK substrate. Dimensional variance should be taken into consideration before printing when setting the dimension compensation parameters. This shrinkage may be tolerable when printing simple shapes, e.g., medical substitutes of mandibular or ribs, when preciseness is not an issue. Regarding small devices with an elaborate design, this imprecision is not acceptable. In conclusion, delicate control of the temperature field in the print area is an important way to reduce deformation and warpage.

**Figure 3:**
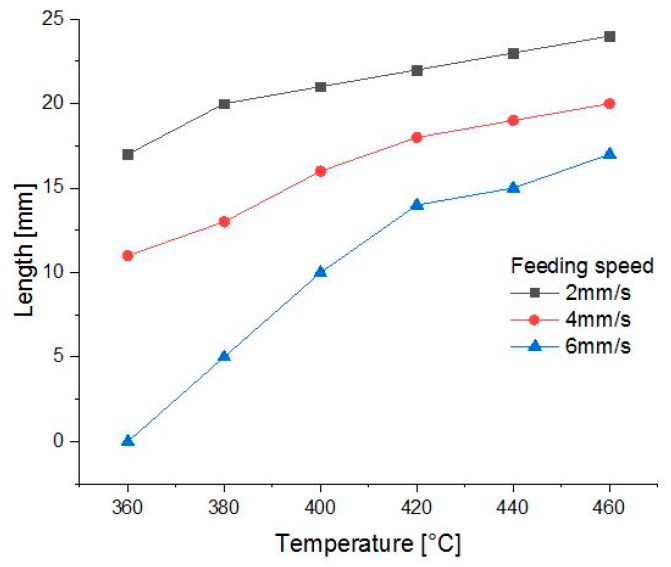
Length of the liquid column in the nozzle with diameter of 0.8 mm using the data from Wang et al. [29].

Viscosity of the material should also be taken into consideration during the FDM procedure. Wang et al. [29] made a finite element analysis (FEA) of PEEK material in the flow channel of the nozzle to explore reasonable printing temperatures. According to their experiment, the heating temperature of the printing head, wire feeding speed, and diameter of the nozzle are the key parameters influencing the distribution of the temperature field, viscosity field, and pressure field. To summarize from their experiment, higher temperature and slower feeding speed guarantee a longer length of the liquid column in the nozzle (Figure 3), and therefore confirm better processing possibility of the material.

#### 4.1.2. Temperature

Nozzle temperature, plate temperature, chamber/ambient temperature, and cooling methods during PEEK printing have a pronounced influence on the crystallization process and therefore affect the properties of finished objects to a large extent [13].

Suitable nozzle temperature can guarantee sufficient time to heat the PEEK filament. If the nozzle temperature is too low, the PEEK filament is not melted completely, which may lead to nozzle obstruction because of the high viscosity of the material. If the nozzle temperature is too high, chain cleavage might occur and possibly leading to decomposition [15]. Nozzle temperatures have been explored within a range from 340 °C to 480 °C [13,15,16,17,18,20,21,22,23]. Wu et al. [15] found when the nozzle temperature is 350 °C, the warping deformation of PEEK samples is minimal. Vaezi et al. [17] identified nozzle temperatures of 400–430 °C as an applicable range. Nozzle temperatures below 400 °C caused either nozzle clogging or delamination of the final product, and above 430 °C resulted in either considerable filament deformation or material degradation. Hu et al. [24] designed a new heater control nozzle module to improve the temperature uniformity in the printing area. They used 385 °C for nozzle temperature in the experiment and reported samples with less warpage and delamination. Wang et al. [29] came up with the conclusion that considering the printing head design and size of the heating block, the length of the nozzle is recommended to be less than 15 mm, and the applicable temperature for PEEK printing should be in the range between 380 °C to 440 °C. Yang et al. [13] applied 360 °C–480 °C in their experiments and used a gradient of 20 °C. Nozzle temperature was found to be a complicated factor for the PEEK’s crystallinity and mechanical properties. When the nozzle temperature increased from 360 °C to 380 °C, the crystallinity first was reduced from 19% to 16%, which can be explained as an incomplete melting of crystalline regions inside the nozzle with a lower and non-uniform temperature. Then, the crystallinity turned into a moderate growth until a relatively stable value (21%) as the nozzle temperature increased up to 480 °C. Higher crystallinity samples performed better mechanical strength, which is shown in Table 2. To sum up, the quality and characteristics of filaments from different companies may account for the difference between these results. The filaments vary from each other so the most suitable printing temperature needs to be explored before the experiment. Taking the two kinds of filaments we applied in our research as examples, filaments made by Evonik can be printed continuously when nozzle temperature is between 420 and 440 °C, while the suitable temperature range for the Apium filaments is 380–400 °C. The reason lies in the purity of the filament. Possible contamination is the filament could be wax, which is brought in during filament manufacture.

Platform temperature, chamber/ambient temperatures, and cooling methods have a direct or indirect impact on the cooling speed of the samples and sequentially affect the crystallinity of the products. Most of the authors used air cooling as cooling methods, while others also explored different cooling methods such as furnace cooling, quenching, annealing, and tempering. Wu et al. [15] came to the conclusion that 130 °C is the most suitable chamber temperature for PEEK printing, while Hu et al. [24] applied 135 °C as plate temperature. Vaezi et al. [17] identified a plate temperature of 130 °C and an ambient temperature of 80 °C as applicable conditions for an extrusion rate of 2.2 mg/s. Han et al. [23] kept the printed PEEK samples in a furnace for 2 h at 300 °C, and let them cool down at room temperature afterward. When the temperature rises above T_g_, there will be a thermodynamic tendency for the polymer to continue to form crystals or to recrystallize [1]. This procedure increased the time for crystalline and may account for the better mechanical performance of the samples. Yang et al. [13] obtained similar results and concluded in their experiment that the higher ambient temperatures would provide more energy and time to improve the crystallinity of PEEK. Furnace and annealing cooling permit samples to stay in temperatures beyond T_g_, causing an isothermal crystallization process, in which the amorphous polymer chains have sufficient energy to transform and crystallize to a degree of around 31%, but still less than the typical crystallinity (35%). In contrast, rapid cooling of PEEK samples, such as quenching and tempering methods, can cause warp distortion, caused by uneven crystallization, because the internal stress leads to significant deformation [13]. Basgul et al. [25] conducted experiments on annealing to seek a possible post-processing method for printed PEEK parts as lumbar spinal cages. They observed that the annealing effect increased the cages’ mechanical properties (14% increases in compression strength) printed with slower speed, indicating annealing might enhance the interlayer adhesion under certain printing conditions. They also concluded that annealing can change the structure of the pores but is not able to decrease the undesired porosity formed during the 3D printing process.

#### 4.1.3. Layer Thickness and Printing Speed

The layer thickness plays key roles in determining the dimensional accuracy and the surface roughness of printed parts [30]. The surfaces of printed objects made by AM exhibit ridges caused inherently by the deposition process. Theoretically, if the layer height is small enough, the surface of the specimens will be smooth. However, the typical minimum feature size obtained with an extrusion AM process is in the order of 100 μm [12]. The authors in the reviewed literature applied different layer heights, which are between 0.1 mm and 0.4 mm. The work of Wu et al. [16] showed that optimal mechanical properties were found in samples with a layer thickness of 0.3 mm. Deng et al. [20] demonstrated that an optimal tensile strength and elongation rate can be achieved when the parameter of layer thickness is 0.25 mm, while the optimal elastic modulus is achieved when the layer thickness is 0.2 mm.

Printing speed is another important factor in 3D printing. If the extrusion speed does not match with the printing speed, there will be extra material sticking to the nozzle causing unstable dimensions of the printed specimens. Geng et al. [22] investigated the effects of the extrusion and printing speed on the microstructure and dimensions of an extruded PEEK filament. They performed the experiments with nozzle diameters of 0.4, 0.5, and 0.6 mm and printing speeds from 0.1 to 120 mm/min. They concluded that during the FDM of PEEK, the melt pressure directly affects the surface morphology and extrusion diameter of the filament, and higher melt pressure is beneficial for the reduction of surface defects on the extruded filament. Rahman et al. [18] took a printing speed of 50 mm/s in their experiments while Han et al. [23] applied a printing speed of 40 mm/s in theirs. Deng et al. [20] achieved optimal tensile properties for PEEK specimens when the printing speed was 60 mm/s. According to the results above, we can assume that a reasonable speed value for the printing of PEEK with a 0.4 mm diameter nozzle should lie in the range of 40–80 mm/s.

**Figure 4:**
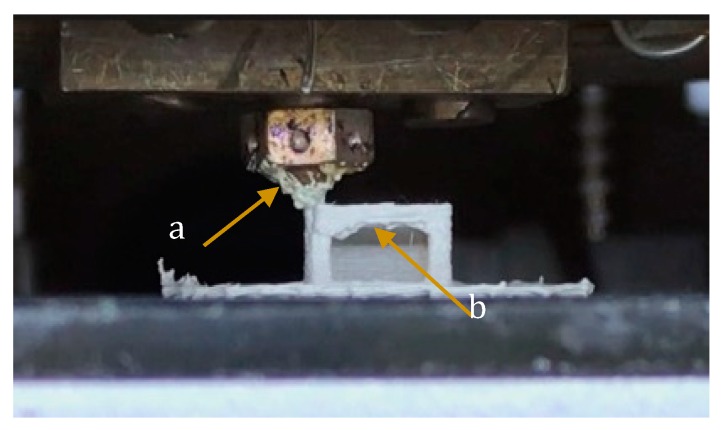
A shot of the 3D printer in the processing of DC4430 PEEK. (**a**) The residual material sticking to the nozzle. (**b**) The structure defect of sample.

The fluctuating extrusion force is the main constraint on the stability of the extrusion process [22]. As shown in Figure 4, the effect of viscosity of the material on extrusion, retraction distance (which means the distance of retraction after printing each layer, usually between 1 and 5 mm), and extrusion multiplier should also be taken into consideration. In short, if the volume of the material extruded from the nozzle is not appropriately synchronized with the volume of material needed for deposition, then it would create either an overflow of polymers or defection of structure flaw of finished parts.

#### 4.1.4. Nozzle Diameter and Nozzle Material

To improve the accuracy of the finished parts, reducing the nozzle diameter seems to be a possible way. Simply decreasing the nozzle diameter will more easily cause blocking of the nozzle and cannot solve the problem of low resolution completely. In the reported literature, nozzles with diameters between 0.2 mm and 0.8 mm are generally applied [18,20,22,23,29]. Wang et al. [29] observed that from the enlarged pressure field of the flow channel, extrusion pressure at the outlet of the nozzle is 10 percent of its original value, as it drops from 5.5 × 10^4^ Pa to 0.45 × 10^4^ Pa, and the diameter of nozzle varies from 0.4 mm to 0.8 mm. A larger diameter nozzle can therefore effectively reduce the wire feeding pressure of the extruder and enlarge the printing layer thickness, which may be favorable for the life of the extruder motor but disadvantageous for the surface quality of the finished part.

The key for accurate manipulation is controlling precisely the outflow of the material, which has to be explored as each individual printing depicts the design of finished objects. However, the principal resolution of the printer software is limited; there is no value to improve the file resolution beyond the recognition of achievable stepper motors in extruded AM systems [30].

Commercially, extrusion printing heads for PEEK printing are commonly made of brass or stainless steel [17]. They are widely used because of their excellent heat-conducting property and resistance to high temperatures. However, metals have a trend of thermal dissipation, which results in the inaccuracy of live temperature during the printing process. Therefore, ceramics might be a possible alternative material as nozzle heads. They have a lower coefficient of thermal expansion and maintain the heat within a certain zone better.

#### 4.1.5. Starting Point and Software

The printing routine/toolpath is one of the key design variables in the production of parts via extrusion-based additive processes [30]. However, in all the reviewed literature concerning PEEK printing, this parameter was not been mentioned as one of the key parameters affecting the quality of samples.

**Figure 5:**
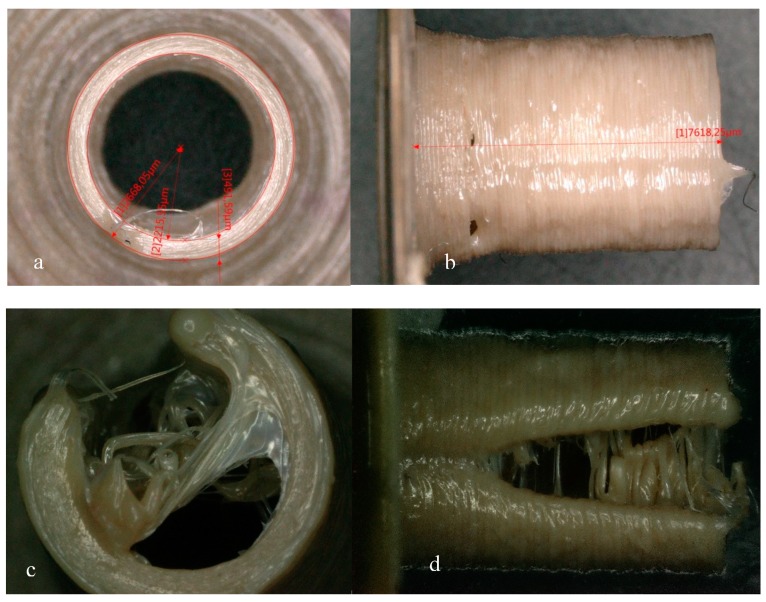
Barrels printed with radius of 3 mm and wall thickness of 0.5 mm by Apium HPP155 with two software. (**a**,**b**) Samples printed by software installed in the printer, and (**c**,**d**) are printed by Simplify 3D, respectively.

To establish a uniform surface, the contour is typically printed around the perimeter of a part. In our experiment, we used two software; one was the printer implemented software and the other was Simplify 3D. When printing a cylinder, samples coded by Simplify 3D software are constructed with reciprocating lines, which means the nozzle is moving clockwise and anticlockwise for one round, and then the printing platform moves up and down to adjust itself for the next layer. On the contrary, software installed in the machine has a different routine. The nozzle moved in one direction all the time, with the platform adjusting its position in the Z-axis almost in the same position. When printing samples with relatively great dimensions—for example, hollow cylinders with an outer radius of 5 mm and a wall thickness of 0.5 mm—there was no significant difference between samples. However, when we reduced the diameter gradually, and approached a radius of 3 mm, the routine used reciprocating lines around one layer had a “V” split line along the starting point (Figure 5). We observed during the printing procedure that the semi-liquid extruded PEEK tended to retract for the starting point because of inner tension; suddenly, a veer of the nozzle dragged the extruded material away. This result may indicate that the back and forth routine has higher requirements for calibration than the mono-directional routine. What is more, we deduce that different slicers may account for this phenomenon, leading to some printing defects in the printing process.

Another interesting phenomenon found in our experiment is that the bottom layers in general have a greater diameter than the upper rim or disk, respectively, resembling an “elephant foot” (Figure 5). This is a popular problem that happened in the FDM strategy. If the bottom layer of the model does not have enough time to cool down during the printing process, then the bottom layer is pressed by the weight of the upper part of the model, which will cause the bottom layer to protrude outward. This situation is more likely to occur especially when the bed temperature setting of the 3D printer is too high. Possible solutions lie in the better calibration of the plate, the fitter distance between the nozzle and plate, as well as a nice setting of plate temperature and cooling fan.

## 5. Own Experiments

A dental implant made of PEEK has better advantages than titanium ones, as it can reduce the risk of allergy and its similar elastic modulus with bone, therefore it can decrease the stress-shielding phenomenon in the site of surgery. Based on the experiences from the literature survey, it was tried to print a dental implant with an inner and outer structure using a commercially available 3D printer for PEEK from Apium (Karlsruhe, Germany) as well as a printer from Orion (Berlin, Germany).

### 5.1. Implant Printed with an Apium HPP155 Printer

**Figure 6:**
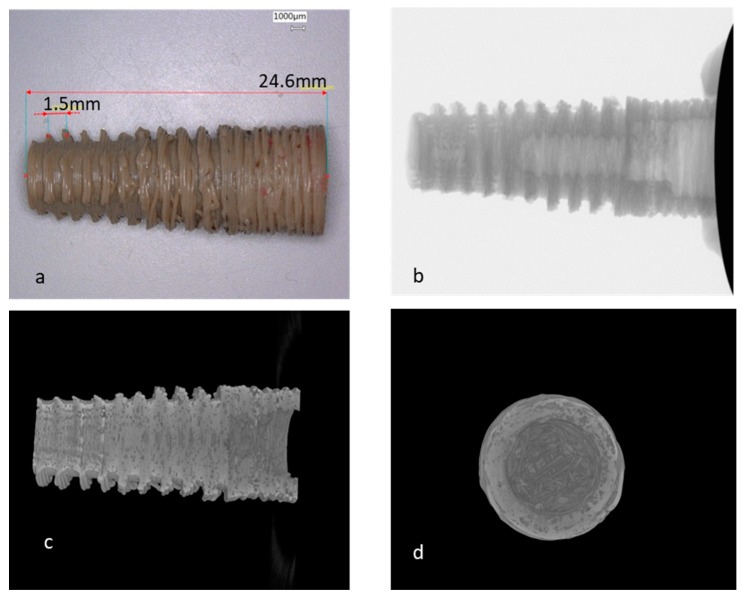
CT of a magnified dental implant produced with Apium HPP155 ((**a**) is the printed implant, (**b**–**d**) are micro CTs of the sample).

We printed a magnified dental implant that is almost three times in dimension of a real one, applying the optimal parameters we got from others’ experience as well as our own trials, to explore the possibility of producing a dental implant with the FDM strategy. As we can see from the image (Figure 6), both the inner and outer screw depth of the implant is not acceptable and the porosity of the sample is evident. The detailed parameters for printing are shown in Table 4. The elastic modulus of the sample is 0.89 GPa, which is 25% of Young’s modulus of the molded PEEK (3.6 GPa). Implants with smaller dimensions were also tried but all of them failed. Since there is no matching substitute nozzle with a smaller nozzle diameter for this printer, we tried to print the implant with another printer from Orion, which was more flexible in nozzle and heater settings.

### 5.2. Implants Printed with Orion Printer

**Figure 7:**
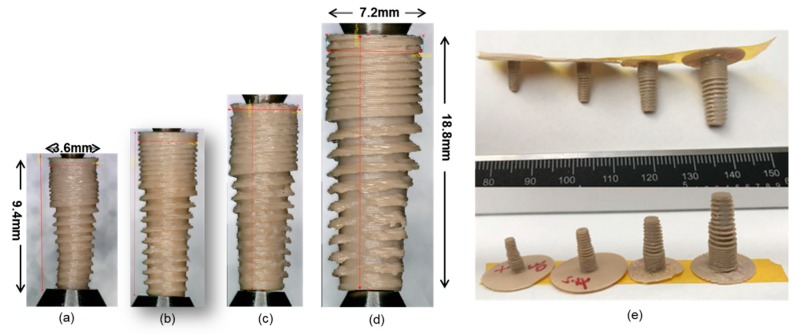
Dental implants printed with Orion Generation 2 ((**b**–**d**) are ×1.2, ×1.5, and ×2 scale of a separately; (**a**,**b**) are printed with a 0.15 mm nozzle at the temperature of 405 °C; (**c**) is printed with a 0.2 mm nozzle at a temperature of 390 °C; (**d**) is printed with a 0.4 mm nozzle at a temperature of 390 °C, (**e**) is the group photo of the above samples).

We printed the same dental implant STL file with the second-generation printer of Orion and got better results, as shown in Figure 7. The smallest successfully printed implant is with a width of 3.6 mm and a length of 9.4 mm, which is a little crooked. We got the best specimens with the 0.15 mm nozzle when printing the ×1.2 scale implant, which is acceptable in both the reproducibility and surface quality. The parameters for printing are shown in Table 5. The elastic modulus of the implants is 2.3 ± 0.28 GPa. This value is within the range of the elastic modulus of cancellous bone suitable for implant surgery, which is between 1.5 GPa and 7.9 GPa [31]. However, smaller nozzles will inevitably lead to longer printing time and are easier to be clogged. This may be ignorable at the research phase, but will increase the cost of manufacturing in mass.

## 6. Conclusions

The patient-orientated therapy model is now drawing efforts to precisely manufactured dental devices and attachments in the dentistry field. Three-dimensional printing is with no doubt an alternative way to accomplish this goal. Considering the great mechanical properties and reliable biocompatibility of PEEK, it has great potential to be used as a substitute for medical devices, and 3D printing strategy offers a promising way of process production and manufacture.

Knowing that for these tests, the last developed machine was not used, we could demonstrate the limits, which drove us to the conclusion that it is necessary to adjust the following parameters. Fr the most widely applied brass nozzle with a diameter of 0.4 mm, its temperature should be maintained within 380 °C–420 °C, the purity of the filament should be taken into consideration, and pre-tests are recommended before the experiment. A constancy of nozzle temperature is better controlled in a range of ±1 degree. Printing speed and retraction speed should be considerately tested considering PEEK types and manufacturers, with the purpose to make the extruded material match with that desired for construction. Further improvement of samples with improved mechanical strength might count on the better solution of 3D printer technology and better manipulation of PEEK.

Until now, the printing of reproducible tiny-sized PEEK parts with high accuracy has proved to be possible in our experiments, which is achieved through optimization of the FDM printing parameters. There is still a long way to go to accomplish the transition from the research phase to 3D printed PEEK manufacturing, and finally to reach the goal of integrating the treatment within clinics. However, this trial might lay a basis for the patient-specialized treatment in the field of dental implantology. Considering the complexity of chewing forces, systematical mechanical tests are needed, and simulation based on finite element analysis is necessary for further research.

## Figures and Tables

**Table 1:** Summary of articles discussing printing parameters and results.

Author	Year of Publication	Materials	Printing System	Parameters	Details	Characterization Techniques
Wu et al. [15]	2014	Unfilled PEEK	Custom-made PEEK 3D printing system	Desiccation	130 °C for 8 h	SEM; 3D scanner
				Nozzle temperature	340 °C–360 °C	
				Chamber temperature	90 °C–130 °C	
Wu et al. [16]	2015	Unfilled PEEK and ABS plusTM-P430	Custom-made PEEK 3D printing system	Layer thickness	0.2, 0.3, 0.4 mm	Tensile, bending, and compressive test
				Raster angle	0°, 30°, 45°	
Vaezi et al. [17]	2015	PEEK OPTIMA LT3 power (Invibo) and Victrex® PEEK 450 G filament	Syringe-based and filament-based extrusion system	Desiccation	150 °C for 3 h	Tensile test, three-point flexural test and compressive test
				Nozzle material	Brass and stainless steel	
				Nozzle temperature	350–450 °C	
				Plate temperature	100 °C	
				Ambient temperature	80 °C	
				Layer thickness	0.2 mm	
Rahman et al. [18]	2016	A proprietary PEEK formulation from Arevo Labs	Arevo Labs 3D printer	Nozzle temperature	340 °C	Tensile, compression, flexural, and impact testing
				Platform temperature	230 °C	
				Nozzle diameter	Approx. 0.2 mm	
				Printing speed	50 mm/s	
				Infill ratio	100%	
				Layer height	0.25 mm	
				Raster orientation	0°, 90°	
Yang et al. [13]	2017	PEEK filament reprocessed from the PEEK pellets (450 G, VICTREX Corp. in UK)	Temperature-control 3D printing system	Nozzle temperature	360, 380, 400, 420, 440, 460, 480	Tensile test, DSC
				Ambient temperature	25, 50, 100, 150, 200	
				Heat treatment methods	Air cooling, furnace cooling, quenching, annealing, tempering	
				Other parameters	Nozzle diameter = 0.4 mm; layer thickness = 0.2 mm; printing speed = 40 mm/s; raster angle—consistent with longest edge	
Berretta et al. [19]	2017	PEEK 450, 1% CNT PEEK 450 and 5% CNT PEEK 450 (filaments diameter = 2.7 ± 0.3 mm)	A MendleMax v2.0 (Maker’s Tool Works)	Desiccation	150 °C for 5 h	Tensile test, short beam shear stress test, SEM, TEM, CT, DSC
				Nozzle temperature	350, 365, 380 °C	
				Layer height	0.2 mm	
Deng et al. [20]	2018	PEEK-1000 bar	Custom-built FDM equipment	Printing temperature	350–370 °C	Tensile, impact, and three-point bending test
				Layer thickness	0.2, 0.25, 0.3 mm	
				Printing speed	20, 40, 60 mm/s	
				Filling ratio	20%, 40%, 60%	
Cicala et al. [21]	2018	Industrial-grade PEEK (Luvocomm, Hamburg, Germany) and PC	Roboze one 400+ (Roboze, Bari, Italy) Stratasys Fortus® 400 mc	Desiccation	140 °C for 48 h (pellet)	Tensile test
				Other parameters	Nozzle temperature = 420 °C; bed temperature = 110 °C; layer height = 0.1 mm; print speed = 20 mm/s; infill = 75%	
Geng et al. [22]	2019	PEEK 450 G VICTREX®	Self-made PEEK 3D printing system	Desiccation	120 °C for 12 h	Surface morphology
				Nozzle diameter	0.4, 0.5, 0.6 mm	
				Nozzle temperature	360 °C	
				Extrusion speed	0.1 to 120 mm/min	
Han et al. [23]	2019	Pure PEEK and carbon-fiber-reinforced PEEK (CFR-PEEK)	3D printer (Jugao-AM Tech. Corp, Xian, China)	Surface modification	Untreated, polished, sandblasted	① Mechanical characterization—tensile, bending, and compressive tests; ② Biological tests—cytotoxicity and cell adhesion test; ③ SEM, surface topography, water contact angle
				Furnace cooling down	300 °C for 2 h in furnace then under room temperature	
				Other parameters	Nozzle diameter = 0.4 mm; nozzle temperature = 420 °C; ambient temperature = 20 °C; layer thickness = 0.2 mm; printing speed = 40 mm/s; raster angle—consistent with the longest edge	
Hu et al. [24]	2019	PEEK filament (Sting3D Technology Co. Ltd.)	Modified printer from Speedy Maker Company	Raster angle	Tensile and bending samples—consistent with longest edge; warpage sample—45°	Warpage measurement, tensile test, and bending test
				Other parameters	Nozzle temperature = 385 °C; nozzle diameter = 0.4 mm; layer thickness = 0.1 mm; printing speed = 25 mm/s; infill ratio = 100%	
Basgul et al. [25]	2019	PEEK OPTIMA™ LT1 (Invibio Biomaterial Solutions Ltd., Thornton Cleveleys, UK)	Indmatec HPP 155/Gen2: Apium Additive Technologies GmbH, Karhlsruhe, Germany	Printing speed	1500, 2000 mm/min	Compression, compression-shear, and torsion tests
				Annealing temperature	200, 300 °C	
				Other parameters	Nozzle diameter = 0.4 mm; nozzle temperature = 390–410 °C; bed temperature = 100 °C; layer thickness = 0.1 mm; infill pattern-rectangle; infill ratio = 100%	
Li et al. [26]	2019	PEEK (grade: ZYPEEK 550 G)	FUNMAT HT FDM 3D printer (INTAMSYS, Shanghai, China)	Parameters	Nozzle diameter = 0.4 mm	Flexural and bending test, DSC, and X-ray μ-CT
					Nozzle temperature = 400 °C	
					Ambient temperature = 90 °C	
					Platform temperature = 160 °C	
					Nozzle moving speed = 15 mm/s	
					Layer thickness = 0.1 mm	
					Raster angle = +45°/−45°	
					Air gap = 0.18 mm	

**Table 2:** Influence of PEEK processing parameters on mechanical properties.

Author	Year	Most Significant Parameters	Tensile Test		Bending Test		Compression Test	
			Tensile Strength	Tensile Modulus	Bending Strength	Bending Modulus	Compressive Strength	Compression Modulus
			(Mpa)	(Gpa)	(Mpa)	(Gpa)	(Mpa)	(Gpa)
Wu et al. [16]	2015	Layer thickness = 300 μm, raster angle = 0/90°	56.60		56.10		60.90	
Vaezi et al. [17]	2015	100% infill rate PEEK	75.06					
		/			132.37	2.43	102.38	
Rahman et al. [18]	2016	Infill = 100%; layer height = 0.25 mm; extruder temperature = 340 °C; platform temperature = 230 °C; printing speed = 50 mm/s; raster angle = 0°	73.00	2.6–2.8	111.70	1.8–1.9	80.90	2.00
Yang et al. [13]	2017	Nozzle temperature = 420 °C	59.00	3.10				
		Cooling method—annealing	81.50	3.90				
		Ambient temperature = 150 °C	85.00	3.90				
Berretta et al. [19]	2017	PEEK 450 G (380 °C)	90.00					
		1% CNT PEEK 450 G (365 °C)	90.00					
		5% CNT PEEK 450 G (350 °C)	94.00					
Cicala et al. [21]	2018	/	69.04 ± 7.01	3.53 ± 0.01				
Deng et al. [20]	2018	Printing speed = 60 mm/s; layer thickness = 0.25 mm; printing temperature = 370 °C; filling rate = 60%	40 ± 4.4	0.50				
Han et al. [23]	2019	PEEK	95.21 ± 1.86	3.79 ± 0.27	140.83 ± 1.97	3.56 ± 0.13	138.63 ± 2.69	2.79 ± 0.11
		CFR-PEEK	101.41 ± 4.23	7.37 ± 1.22	159.25 ± 13.54	5.41 ± 0.51	137.11 ± 3.43	3.51 ± 2.12
Li et al. [26]	2019	PEEK			146 ± 3.3	3.44 ± 0.05		
		CF-PEEK			146 ± 4.2	3.74 ± 0.09		
Hu et al. [24]	2019	PEEK	74.70	1.15	120.20	1.15		
Reference	/	VICTREX^®^ PEEK 450 G molded PEEK	98.00	4.00	165.00	3.80	125.00	3.80

**Table 3:** Some performance of unfilled PEEK important for 3D printing.

Performance		Testing Method/Standard	Value	Reference
Density	Crystalline	ASTM D792	1.32 g/cm^−3^	[1]
Typical crystalline ratio		N/A	35%	[13]
Melting temperature (T_m_)		DSC	343 °C	[1]
Glass transition temperature (T_g_)		DSC	143 °C	[1]
Coefficient of thermal expansion (CTE)	<T_g_	ASTM D696	5.5 × 10^−5^ K^−1^	VICTREX^®^
	>T_g_		14.0 × 10^−5^ K^−1^	
Heat deflection temperature (HDT)		ASTM D648	152 °C	VICTREX^®^

**Table 4:** The parameters used for dental implant printed with Apium HPP155 printer.

Parameters	Value
Filament diameter	1.75 mm
Nozzle diameter	0.4 mm
Nozzle temperature	395 °C
Printing speed	13 mm/s
Layer thickness	0.1 mm
Slicer	Machine-installed slicer

**Table 5:** The parameters used for a ×1.2 scale dental implant printed with the second-generation printer of Orion.

Parameters	Value
Filament diameter	1.75 mm
Nozzle diameter	0.15 mm
Nozzle temperature	405 °C
Chamber temperature	250 °C
Plate temperature	250 °C
Layer heater	200 °C
Printing speed	400 mm/min
Layer thickness	0.05 mm
Slicer	Simplify 3D
Printing time	49 m 12 s
